# Association between multi-metal co-exposure and thyroid cancer risk in Shanxi, China: A case-control study

**DOI:** 10.1371/journal.pone.0334872

**Published:** 2026-01-23

**Authors:** Yifan Xie, Yuanzheng Ding, Yuli Gao, Ke Yang, Yubo Zhang, Lei Li, Ying Wang, Jin Zhang, Shujing Li, Liangpo Liu, Yi Gao, Jing Liu

**Affiliations:** 1 Academy of Medical Sciences, Shanxi Medical University, Taiyuan, China; 2 The First Clinical Medical College of Shanxi Medical University, Taiyuan, China; 3 School of Public Health, MOE Key Laboratory of Coal Environmental Pathogenicity and Prevention, Shanxi Medical University, Taiyuan, China; 4 Department of Thyroid Surgery, The First Hospital of Shanxi Medical University, Taiyuan, China; King Abdulaziz University Faculty of Medicine, SAUDI ARABIA

## Abstract

Environmental metals are established thyroid carcinogens, yet how their co-exposure collectively reshapes carcinogenic mechanisms remains elusive. This study used ICP-MS to quantify plasma concentrations of 18 metals in 368 thyroid cancer patients and matched controls, with free thyroxine (FT4) levels abstracted from medical records. Subsequently, we applied integrative statistical modeling comprising WQS regression, BKMR modeling, interaction analysis, and causal mediation analysis to elucidate exposure-response relationships and mediating pathways. Cases exhibited elevated plasma levels of Cr, Co, Mn, Ni, Cu, As, Cd, and Sn. WQS regression prioritized Sn, Cd, Ni, and As as risk-contributing metals demonstrating positive weight values, while Sb, Pb, and Zn showed inverse associations. BKMR modeling visualized exposure-response relationships, indicating elevated thyroid cancer risks at higher quantiles of co-exposure. A critical synergistic interaction was identified between the Cd-Sn pair. Causal mediation analysis confirmed FT4 mediates 46–69.3% of thyroid cancer risk for Sn, Cd, Ni, and As. Collectively, these findings highlight the need to incorporate metal mixture surveillance and FT4-based endocrine pathway screening into precision prevention frameworks.

## 1. Introduction

Recognized as the most prevalent endocrine malignancy, thyroid cancer accounts for over 40,000 deaths annually [1]. By 2030, there will be the second most common cancer in women and the ninth in men [2]. While the etiology is not completely understood, researchers have implicated radiation exposure, genetic mutations, and dysregulation of thyroid function, specifically elevated TSH levels and high levels of free thyroxine (FT4), as key contributors [3,4]. Given the established role of thyroid hormone dysregulation, researchers have increasingly substantiated the role of environmental exposures, particularly metal contamination, as primary etiological contributors that may disrupt thyroid homeostasis [5]. These harmful substances enter the human body mainly in three ways: by eating or drinking contaminated food and water, breathing in metal particles from the air, and being absorbed through the skin [6]. Therefore, this study focuses on Shanxi Province, a region with documented evidence linking environmental metal exposure to adverse health outcomes in its local population [7]. Such exposure is of particular concern in this region as toxic metals can interfere with thyroid hormone synthesis, metabolism, and signaling in specific ways. For instance, Pb can block thyroxine from binding to transport proteins, potentially elevating free hormone levels like FT4, while Cd may inhibit thyroid-stimulating cell function [8]. Such disruptions could contribute to the observed associations between FT4 levels and cancer risk. Population-based studies have systematically validated several metals, including Cr, Mn, Ni, Cd, Sb, and Tl, as thyroid carcinogens [9]. Meta-analytic evidence positions Se and Mg as protective agents, while Cu demonstrates risk-enhancing properties [10]. Interestingly, studies examining progression risk show associations between higher levels of Cr, As, and Pb and increased risk, while Li, Cu, Zn, and Co appear protective in this context [11].

Given inconsistencies in prior methods and findings and the paucity of studies on multi-metal co-exposure effects in thyroid cancer, this investigation employed an integrated analytical framework. WQS regression was applied to identify driver metals and quantify the joint effect of the mixture. BKMR modeling was utilized to characterize complex nonlinear and non-additive interactions among metals. Synergistic interactions between specific metal pairs were evaluated, and Causal mediation analysis was conducted to quantify the potential mediating role of FT4 in the relationships between metal exposures and thyroid cancer risk. Collectively, this multi-method approach elucidates thyroid cancer risks from metal co-exposure, providing actionable evidence for targeted prevention.

## 2. Method

### 2.1. Research design

Clinical specimens were collected from thyroid cancer patients pathologically confirmed and surgically treated at the Thyroid Surgery Department of the First Hospital of Shanxi Medical University from June 1 to September 30, 2024. Healthy controls were recruited from the same hospital’s health examination center. Case and control groups were individually matched 1:1 by sex and age (±2 years). The final inclusion of 368 TC cases and 368 matched controls was decided after thoroughly reviewing clinical records, pathology reports, questionnaire answers, and lab test results. Healthy control subjects were enrolled from the health examination center of the same hospital. The study protocol was approved by the Ethics Committee of the First Hospital of Shanxi Medical University (No. KYLL-2024–119). Written informed consent was obtained from all participants for both study participation and the publication of their anonymized data.

#### 2.1.1. Sample size estimation.

The sample size was estimated using the standard formula for 1:1 matched case-control studies [12]. The calculation was based on a conservative odds ratio (OR) of 2.0, a metal exposure rate of 13% in the control population, a significance level of 0.05 (two-sided), and 90% statistical power. This yielded a minimum requirement of 308 case-control pairs. To ensure robust power, we recruited 368 pairs, which also provides adequate power to detect more modest effect sizes. The standard formula used for this calculation is provided in Supplementary S1 Fig.

#### 2.1.2. Inclusion and exclusion criteria.

The inclusion criteria included: (1) patients who were pathologically confirmed with thyroid carcinoma; (2) adults who were 18 years or older; (3) those who provided written consent and completed standard questionnaires; (4) those who had complete medical records, including their medical history and treatment details. Exclusion criteria encompassed: (1) individuals diagnosed with other malignancies; (2) patients with severe hepatic/renal dysfunction; (3) those who were documented metal exposure histories (occupational/environmental) within the preceding 12 months; (4) pregnant or lactating women.

### 2.2. Data collection

A self-designed questionnaire collected participant information spanning demographic characteristics—specifically age, sex, educational attainment, and monthly household income—alongside health status indicators including BMI, hypertension, diabetes and dyslipidemia history. Lifestyle factors encompassed smoking status, alcohol consumption patterns, and exercise frequency, with additional documentation of family thyroid cancer histories. Pathological test results comprising FT3, FT4, and TSH levels, as well as lymph node metastasis status, were retrieved from hospital information systems.

### 2.3. Sample collection and processing

Within 12 hours of hospital admission, we collected whole blood samples from each thyroid cancer patient. After collection, samples were immediately centrifuged for plasma separation. The isolated plasma was stored at a −80°C environment until subsequent processing and analysis. Metal quantification was conducted using a validated methodology previously established in prior research (7).

#### 2.3.1. Plasma sample treatment.

Frozen plasma samples were initially thawed at 4°C. Subsequently, aliquots were mixed with concentrated nitric acid and hydrogen peroxide and subjected to digestion in a 100°C water bath for 8 hours. Following digestion, samples were diluted 20-fold with ultrapure water prior to metal concentration analysis.

#### 2.3.2. Metal detection.

Plasma concentrations of 18 metals (Al, As, Cd, Co, Cr, Cu, Fe, Mg, Mn, Ni, Pb, Sb, Se, Sn, Ti, Tl, V, Zn) were analyzed using inductively coupled plasma mass spectrometry (ICP-MS; Agilent 7700x). Calibration curves were established according to the concentration ranges of individual metals and the detection results of sample supernatants. For metal concentrations below the LOD, values were substituted with LOD/√2, a common practice in environmental epidemiology to reduce bias in statistical analyses. Due to low detection rates for thallium (Tl), this element was excluded from subsequent analyses. See S1 Table for more details regarding the limits of detection and limits of quantitation.

### 2.4. Statistical analysis

Normally distributed continuous variables were expressed as mean ± standard deviation, while non-normally distributed variables were presented as median (interquartile range). Categorical variables were reported as frequencies and percentages. Plasma metal concentrations underwent natural logarithm (ln) transformation to approximate normality. Between-group comparisons of continuous variables were performed using t-tests or Mann-Whitney U tests based on data distribution, and chi-square tests were applied for categorical variables. To account for multiple comparisons across the seventeen metals analyzed, the False Discovery Rate (FDR) was controlled using the Benjamini-Hochberg procedure. All reported significant associations survived this correction (FDR-adjusted p < 0.05). The Spearman correlation coefficient was initially employed to assess pairwise correlations between metals. Subsequently, metal mixture effects were evaluated using WQS regression, which assigned differential component importance through 10,000 repeated samplings. To further characterize complex relationships, BKMR modeling with Markov Chain Monte Carlo algorithms (25,000 iterations) was implemented, specifically capturing nonlinear and non-additive interactions. Convergence of the BKMR model was assessed by visual inspection of the trace plots, which demonstrated satisfactory mixing and stationarity. Concurrently, interaction analysis using generalized linear models (GLM) with binomial distributions and logit link functions investigated synergistic effects between factors, while causal mediation analysis systematically elucidated mechanistic pathways linking metal exposure to thyroid cancer. All statistical analyses were conducted in R (version 4.4.1).

## 3. Results

### 3.1. Baseline characteristics of thyroid cancer group and control group

This study comprised 368 thyroid cancer cases and 368 matched controls, with Table 1 summarizing the demographic and clinical characteristics of the study cohort. The case and control groups differed significantly in all characteristics except for age and gender (P < 0.05).

**Table 1:** Baseline characteristics of thyroid cancer and control group.

Characteristic	Overall (N = 736)	Control (N = 368)	Case (N = 368)	*P*-value
Gender				>0.9
Female	554 (75%)	277 (75%)	277 (75%)	
Male	182 (25%)	91 (25%)	91 (25%)	
Age (years)	46 ± 12	46 ± 12	46 ± 12	0.6
BMI(kg/m^2^)	23.9 ± 4.6	22.1 ± 4.9	25.6 ± 3.6	<0.001
Education Level				<0.001
Primary	67 (9.1%)	32 (8.7%)	35 (9.5%)	
Junior	211 (29%)	105 (29%)	106 (29%)	
Senior	230 (31%)	91 (25%)	139 (38%)	
College	228 (31%)	140 (38%)	88 (24%)	
Family Income				<0.001
< 3000	161 (22%)	77 (21%)	84 (23%)	
3000-6000	334 (45%)	193 (52%)	141 (38%)	
> 6000	241 (33%)	98 (27%)	143 (39%)	
Hypertension				<0.001
No	639 (87%)	342 (93%)	297 (81%)	
Yes	97 (13%)	26 (7.1%)	71 (19%)	
Diabetes				0.011
No	675 (92%)	328 (89%)	347 (94%)	
Yes	61 (8.3%)	40 (11%)	21 (5.7%)	
Hyperlipidemia				0.007
No	662 (90%)	342 (93%)	320 (87%)	
Yes	74 (10%)	26 (7.1%)	48 (13%)	
Smoking				<0.001
No	664 (90%)	347 (94%)	317 (86%)	
Yes	72 (9.8%)	21 (5.7%)	51 (14%)	
Alcohol				0.035
No	643 (87%)	331 (90%)	312 (85%)	
Yes	93 (13%)	37 (10%)	56 (15%)	
Physical Activity				<0.001
< 7h/week	497 (68%)	323 (88%)	174 (47%)	
7-10h/week	133 (18%)	45 (12%)	88 (24%)	
>=10h/week	106 (14%)	0 (0%)	106 (29%)	
Family history of TC				<0.001
No	719 (98%)	368 (100%)	351 (95%)	
Yes	17 (2.3%)	0 (0%)	17 (4.6%)	

Note: Continuous variables are presented as mean ± standard deviation; categorical variables are presented as n (%).

### 3.2. Plasma metal levels

**Fig 1:**
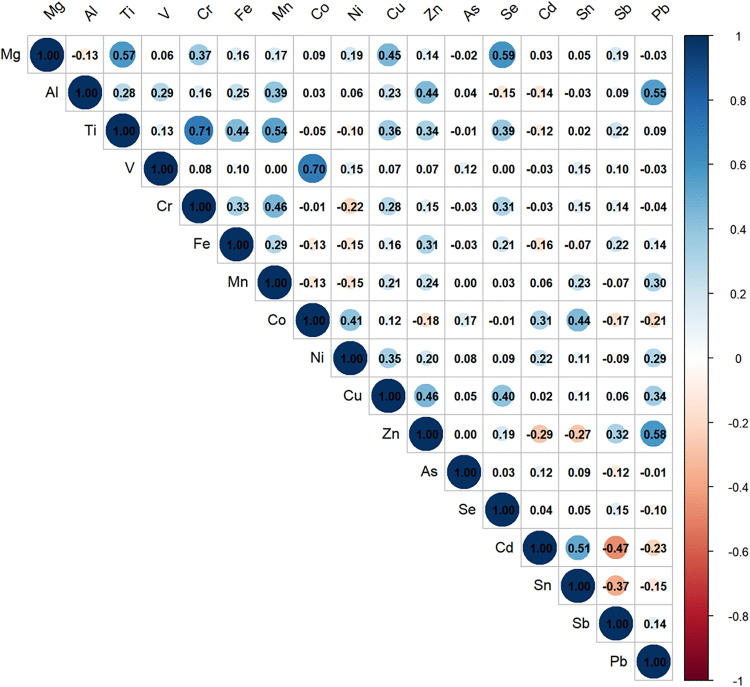
Spearman correlation analysis of Plasma metals.

Significantly lower plasma concentrations of Al, Ti, Fe, Zn, Sb, and Pb (p < 0.05) were observed in the case group compared to controls, whereas elevated levels of Cr, Co, Mn, Ni, Cu, As, Cd, and Sn (p < 0.05) were detected (Table 2).Spearman’s correlation analysis was performed on Plasma metals, showing that Ti and Cr had the strongest correlation (r = 0.71), with other metals displaying moderate to weak correlations (r = −0.47–0.7), as detailed in Fig 1.

**Table 2:** Quartiles of Plasma metal.

Metals	Quartiles of Plasma metal (µg/L)		*P*-value
	Control(N = 368)	Case(N = 368)	
Mg	1,376 (1,205, 1,497)	1,375 (1,244, 1,523)	0.12
Al	21 (15, 36)	18 (15, 23)	<0.001
Ti	7.81 (7.12, 8.96)	7.35 (6.84, 7.98)	<0.001
V	0.14 (0.07, 0.30)	0.15 (0.13, 0.18)	0.2
Cr	9.18 (8.82, 9.79)	9.67 (9.37, 10.00)	<0.001
Fe	95 (61, 133)	73 (56, 95)	<0.001
Co	0.03 (0.02, 0.13)	0.10 (0.09, 0.13)	<0.001
Mn	0.47 (0.41, 0.55)	0.61 (0.53, 0.72)	<0.001
Ni	1.76 (1.62, 1.88)	1.88 (1.81, 1.97)	<0.001
Cu	48 (42, 55)	50 (43, 56)	0.017
Zn	80 (67, 98)	63 (56, 73)	<0.001
As	0.12 (0.09, 0.16)	0.18 (0.12, 0.23)	<0.001
Se	4.40 (3.72, 5.09)	4.39 (3.73, 5.10)	>0.9
Cd	0.26 (0.21, 0.28)	0.51 (0.40, 0.55)	<0.001
Sn	0.11 (0.07, 0.18)	0.62 (0.37, 0.81)	<0.001
Sb	0.52 (0.41, 0.64)	0.26 (0.18, 0.36)	<0.001
Pb	0.48 (0.34, 0.83)	0.22 (0.15, 0.41)	<0.001

Note: Data are presented as median (interquartile range) in µg/L.

### 3.3. Association between plasma metal concentrations and risk of thyroid cancer

Multiple statistical approaches were systematically implemented to assess associations between plasma metal concentration and thyroid cancer risk, including WQS regression, BKMR analysis, interaction effect evaluation, and causal mediation analysis. All models were adjusted for potential confounders: BMI, smoking status, alcohol consumption, exercise frequency, monthly household income, educational attainment, diabetes, dyslipidemia, hypertension, FT3, FT4, TSH levels, lymph node metastasis, and family history of thyroid disorders.

#### 3.3.1. WQS regression analysis.

**Fig 2:**
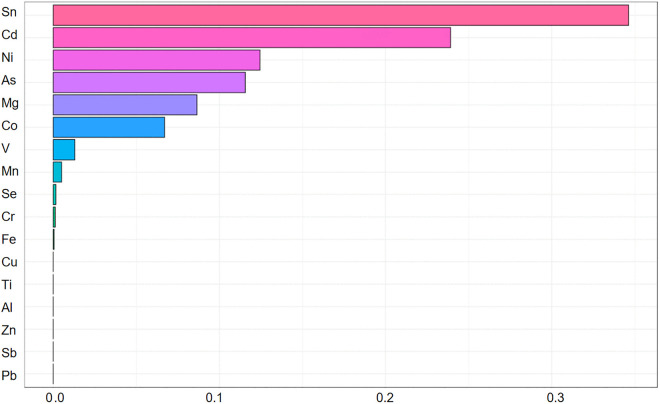
Positive WQS weight distribution map.

**Fig 3:**
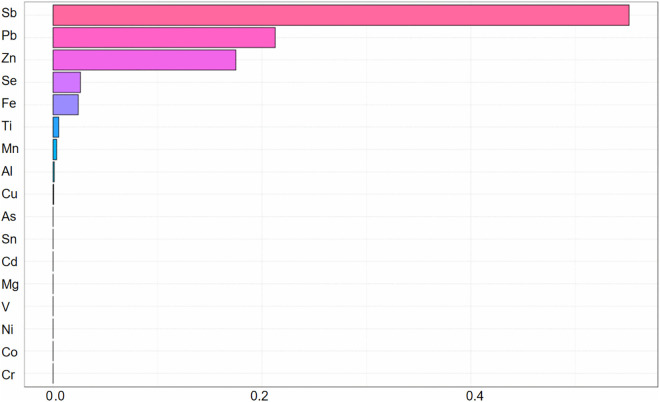
Negative WQS weight distribution map.

We implemented WQS regression to evaluate the combined effects of metal mixtures on thyroid cancer risk. In the positive directional WQS model (Fig 2), Sn, Cd, Ni, and As exhibited positive weights for thyroid cancer risk, with weights of 0.346, 0.239, 0.124 and 0.115. This mixture demonstrated a significant positive association with thyroid cancer (OR = 1.69, 95% CI: 1.63–1.75; p < 0.001). In the negative directional WQS model (Fig 3), Sb, Pb, and Zn exhibited negative weights for thyroid cancer risk, with weights of 0.551, 0.213, and 0.175. Exposure to this protective mixture was inversely associated with thyroid cancer risk (OR = 0.72, 95% CI: 0.69–0.76; p < 0.001). The overall joint effect of the mixture estimated by the WQS regression was statistically significant (p < 0.001), indicating a good fit of the model.

#### 3.3.2. BKMR modeling analysis.

**Fig 4:**
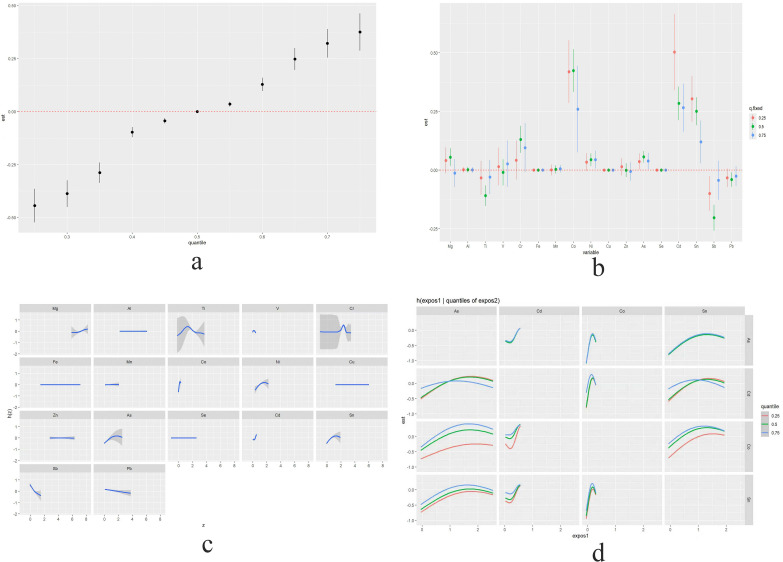
BKMR analysis of metal mixture effects on thyroid cancer risk. **a.** Cumulative effect of metal mixture; **b.** Single metal combined effect; **c.** Single variable exposure-response function for each metal; **d.** Bivariate exposure-response function associated with mixed metal exposure and thyroid cancer.

We implemented Bayesian Kernel Machine Regression (BKMR) to evaluate the exposure-response relationships. The model revealed a progressive increase in thyroid cancer risk with higher exposure quantiles, as visualized in Fig 4a. The overall odds ratio (OR) for the 75th percentile compared to the 50th percentile was 2.47 (95% CI: 1.36–4.49), indicating a statistically significant increase in risk at higher exposure levels.

To assess independent metal effects, we employed a quantile-based g-computation approach. When increasing target metals from the 25th to 75th percentiles while fixing others at 25th/50th/75th percentiles, elevated concentrations of Co, As, Cd, and Sn consistently showed significant positive associations with thyroid cancer risk across all fixed thresholds. Notably, effect sizes exhibited non-monotonic patterns across thresholds, suggesting complex metal interactions or population heterogeneity in exposure responses (Fig 4b).

Linear exposure-response relationships were observed for Mg (positive association), Sb, and Pb (both inverse associations) (Fig 4c). Pairwise interaction analysis further identified significant interactions between Cd-Sn, Cd-As, Cd-Sb, Cd-Mg, and Mg-Co (Fig 4d, S2 Fig).

#### 3.3.3. Interaction analysis.

**Fig 5:**
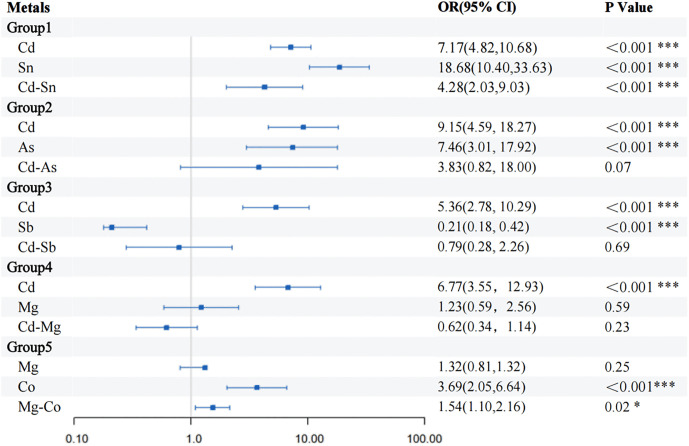
Forest diagram of the relationship between metal-to-metal interactions and thyroid cancer. * P < 0.05, ** P < 0.01, and *** P < 0.001.

The forest plot evaluated the effects of five metal interaction pairs (Cd-Sn, Cd-As, Cd-Sb, Cd-Mg, Mg-Co) on thyroid cancer risk. Results indicated that only Cd-Sn (P ＜ 0.001, OR = 4.28, 95% CI: 2.03–9.03) and Mg-Co (P = 0.02, OR = 1.54, 95% CI: 1.10–2.16) exhibited statistically significant interaction effects, suggesting that these pairs may enhance thyroid cancer risk through synergistic mechanisms (Fig 5). In contrast, no significant interactions were observed for the remaining pairs (Cd-As, Cd-Sb, Cd-Mg).

#### 3.3.4. Causal mediation analysis.

**Fig 6:**
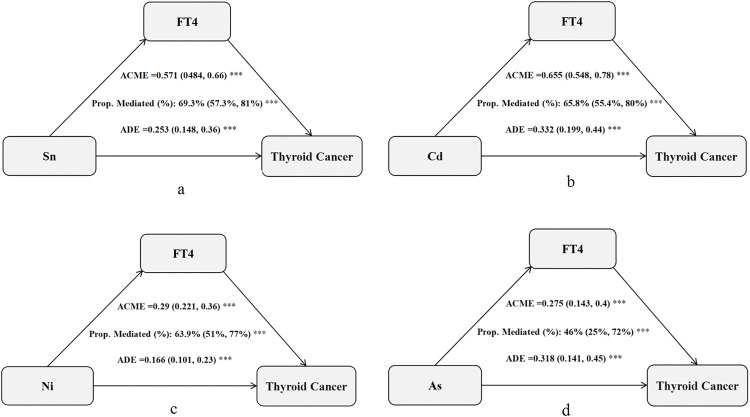
Pathway analysis of the mediating effect of metals on the risk of thyroid cancer. **a.** Analysis of the mediating effect pathway between Sn and thyroid cancer; **b.** Analysis of the mediating effect pathway between Cd and thyroid cancer; **c.** Analysis of the mediating effect pathway between Ni and thyroid cancer; **d.** Analysis of the mediating effect pathway betweenAs and thyroid cancer. Notes: ACME, average causal mediation effects (indirect effect); ADE, average direct effects. * P < 0.05, ** P < 0.01, and *** P < 0.001.

Significant mediation effects of FT4 were observed in the relationships between four metals (Sn, Cd, Ni, As) and thyroid cancer risk (Fig 6). The mediated proportions (%) of FT4 in the associations of Sn, Cd, Ni, and As with thyroid cancer were 69.3% (95% CI: 57.3%, 81%), 65.8% (95% CI: 55.4%, 80%), 63.9% (95% CI: 51%, 77%), and 46% (95% CI: 25%, 72%), respectively. These results suggest that FT4 partially explains the biological pathways linking these metals to thyroid cancer.

#### 3.3.5. Sensitivity analysis.

To assess the robustness of our primary findings, we conducted comprehensive sensitivity analyses by comparing fully adjusted models with models adjusting for age and sex only. For the main metal associations, the significant relationships identified for key metals (including Cd, Sn, Ni, and As) remained stable across model specifications, with consistent effect directions and magnitudes. For interaction effects, the synergistic interaction between cadmium and tin (Cd-Sn) remained statistically significant and stable. In contrast, the interaction between magnesium and cobalt (Mg-Co) was attenuated and lost statistical significance in the sensitivity analysis, indicating that this finding requires cautious interpretation.

## 4. Discussion

Employing an integrated analytical framework of WQS regression, BKMR, interaction assessment, and mediation modeling, our study demonstrates that environmental metal mixtures collectively elevate thyroid cancer risk through three interconnected pathways: divergent metal-specific effects encompassing both risk-enhancing metals (Sn, Cd, Ni, As) and protective elements (Sb, Pb, Zn), synergistic interactions between specific metal pair (Cd-Sn), and endocrine disruption mediated predominantly by FT4. These findings establish thyroid cancer as a mixture-dynamic process wherein combinatorial exposure interactions dictate disease risk beyond the summed effects of individual metals, necessitating a paradigm shift in environmental risk assessment.

WQS regression identified Sn and Cd as dominant positive drivers within the metal mixture, significantly elevating thyroid cancer risk (Fig 2). Dietary exposure to Sn occurs primarily through canned foods [13], where its organotin compound tributyltin (TBT) disrupts hypothalamic-pituitary-thyroid (HPT) axis homeostasis by acting on nuclear receptors (RXR/PPARγ), interfering with thyroid hormone synthesis, secretion, and metabolism to ultimately promote thyroid cancer [14]. Cd exposure arises mainly from contaminated food and water, with additional sources including tainted pharmaceuticals and supplements [15]. Functionally mimicking both 17β-estradiol (E2) and the GPER-specific agonist G1, Cd induces proliferation, invasion, and migration in GPER-expressing human thyroid cancer cell lines (WRO and FRO) [16]. This mechanistic evidence aligns with epidemiological observations in volcanic regions, where populations in basaltic environments with elevated environmental Cd levels exhibit higher thyroid cancer incidence than non-volcanic areas [17],and is further corroborated by significantly elevated Cd concentrations in thyroid tissue of cancer patients versus controls [18].

Ni and As also demonstrated positive risk associations. Nickel acts as a ubiquitous endocrine disruptor that directly impairs thyroid hormone homeostasis, contributing to thyroid dysfunction and cancer [9,19]. Conversely, Sb emerged as a protective factor (Fig 3), consistent with prior epidemiological reports of reduced thyroid cancer risk [9]. Zn—critical for thyroid hormone metabolism—showed an inverse association, where deficiency correlates with impaired thyroid function and increased cancer susceptibility [20,21]. This is consistent with previous findings regarding zinc’s protective role [22,23].

The inverse association for Pb presents a paradox. While most studies link this food-system endocrine disruptor [24] to increased risk—particularly in multiparous women [25,26] through HPT-axis disruption [27] and dose-responsive papillary carcinoma relationships [28–30]—our findings suggest potential protective effects. This discordance may reflect cohort-specific characteristics, unmeasured confounders, or threshold effects in Pb toxicity.

BKMR modeling showed that the risk of thyroid cancer increased with higher doses, especially at the higher levels, where a clear positive link was found (Fig 4a). This trend is mechanistically supported by evidence that metal exposure induces reactive oxygen species (ROS) production. ROS generation is recognized as a critical pathway through which DNA, lipids, and proteins are damaged, thereby promoting malignant cellular transformation [31].

Our study identified a significant and robust synergistic interaction between Cd and Sn in elevating thyroid cancer risk (Fig 5), indicating that their co-exposure may amplify carcinogenicity beyond individual effects.. Organotin compounds (OTCs) have been demonstrated to interfere with thyroid axis (HPT axis) regulation [32]. Cd-Sn co-exposure may promote thyroid cancer through endocrine disruption mechanisms by jointly interfering with thyroid hormone homeostasis. In contrast, the interaction between Mg and Co, which was observed in our primary model, was not robust in sensitivity analyses and should be interpreted as a preliminary finding. While Mg acts as an independent protective factor against thyroid cancer [33], experimental evidence indicates that Co exposure suppresses TSH secretion and promotes thyroid dysfunction [20], paradoxically enhancing risk through their interaction. This phenomenon may involve cobalt’s toxic interference in magnesium-dependent biological processes, where Co^2^ ⁺ competitively displaces Mg^2^⁺ in metalloenzymes, causing metal misincorporation that disrupts critical cellular processes and promotes cancer [34]. Notably, the robust Cd-Sn interaction occurred despite only a moderate correlation (r = 0.51), while the Mg-Co interaction, which was statistically unstable, emerged with a negligible correlation (r = 0.09) (Fig 1). This contrast demonstrates that while significant biological interactions can be identified between metals with varying correlation strengths, their statistical robustness must be rigorously evaluated, moving beyond simple correlation methods.

To our knowledge, this represents the first study identifying FT4 as a mediator linking exposure to metals (Sn, Cd, Ni, As) with thyroid cancer. Our analysis demonstrates that FT4 mediates 46–69.3% of these effects (Fig 6)—quantifying a novel endocrine pathway in metal-driven cancer development. Notably, Sn exhibits exceptionally high mediation (69.3%), consistent with its nuclear receptor interference properties. This positions FT4 as the primary conduit for emerging thyrotoxic metals beyond established disruptors like Cd, Ni, and As [35]. Future research should validate the generalizability of these mediation pathways across diverse exposure settings; develop targeted interventions to stabilize thyroid hormone homeostasis; delineate FT4-independent mechanisms underlying partial mediation effects; and investigate how metal mixture interactions amplify endocrine disruption.

This study has limitations. Its single-center design in an industrial region may limit the generalizability of the findings. Exposure misclassification is possible as single plasma measurements may not reflect long-term metal burden, likely biasing results toward the null. Furthermore, the sample size may be underpowered for detecting weak effects or complex interactions, and unmeasured mediators preclude a complete mechanistic understanding.

## 5. Summary

In conclusion, our integrated analyses identify Sn, Cd, Ni, and As as risk-elevating metals for thyroid cancer, while Sb, Pb, and Zn show inverse associations with risk. Critically, the interaction between Cd-Sn exhibited synergistic toxicity, demonstrating that the consequence of this specific co-exposure transcends the additive effects of the individual metals. Causal mediation modeling revealed that FT4 statistically mediates 46–69.3% of the associations between exposure to Sn, Cd, Ni, As and thyroid cancer risk, with Sn exhibiting the highest mediation proportion (69.3%). These findings necessitate integrating multi-metal co-exposure assessment—prioritizing synergistic pairs (Cd-Sn) and endocrine disruption screening via FT4 monitoring—into thyroid cancer prevention programs.

## Supporting information

S1 Fig Sample size calculation formula for the 1:1 matched case-control study. (DOCX)

S2 Fig Bivariate exposure-response function associated with mixed metal exposure and thyroid cancer. (DOCX)

S1 Table Analytical performance of measured metals: limits of detection (LOD), quantitation (LOQ), detection rates, and precision. (DOCX)

## Abbreviations

ICP-MS: inductively coupled plasma mass spectrometry
FT3: Free Triiodothyronine
FT4: Free Thyroxine
TSH: Thyroid-Stimulating Hormone
Al: Aluminum
As: Arsenic
Cd: Cadmium
Co: Cobalt
Cr: Chromium
Cu: Copper
Fe: ferrum
Mg: Magnesium
Mn: Manganese
Ni: Nickel
Pb: Lead
Sb: Antimony
Se: Selenium
Sn: Tin
Ti: Titanium
Tl: Thallium
V: Vanadium
Zn: Zinc
TC: Thyroid Cancer
BMI: Body Mass Index
WQS: Weighted Quantile Sum
BKMR: Bayesian Kernel Machine Regression
