# Supplementary figures and images for: Association between multi-metal co-exposure and thyroid cancer risk in Shanxi, China: A case-control study
Source: PLoS One. 2026 Jan 23;21(1):e0334872. doi: 10.1371/journal.pone.0334872 (PMC12829857; doi:10.1371/journal.pone.0334872)

Supplementary Figure S1. Sample size calculation formula for the 1:1 matched case-control study.


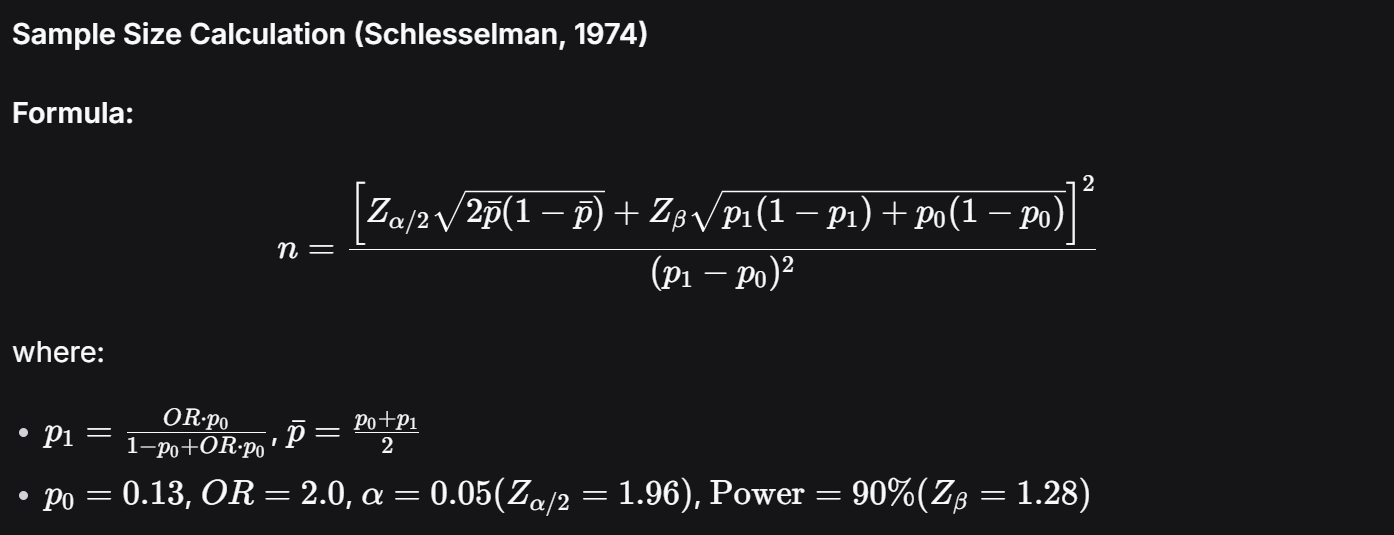

Supplement: S1 Fig — (DOCX) [file pone.0334872.s001.docx]
