# Supplementary material for: Association between multi-metal co-exposure and thyroid cancer risk in Shanxi, China: A case-control study
Source: PLoS One. 2026 Jan 23;21(1):e0334872. doi: 10.1371/journal.pone.0334872 (PMC12829857; doi:10.1371/journal.pone.0334872)
Supplement: S2 Fig — (DOCX) [file pone.0334872.s002.docx]

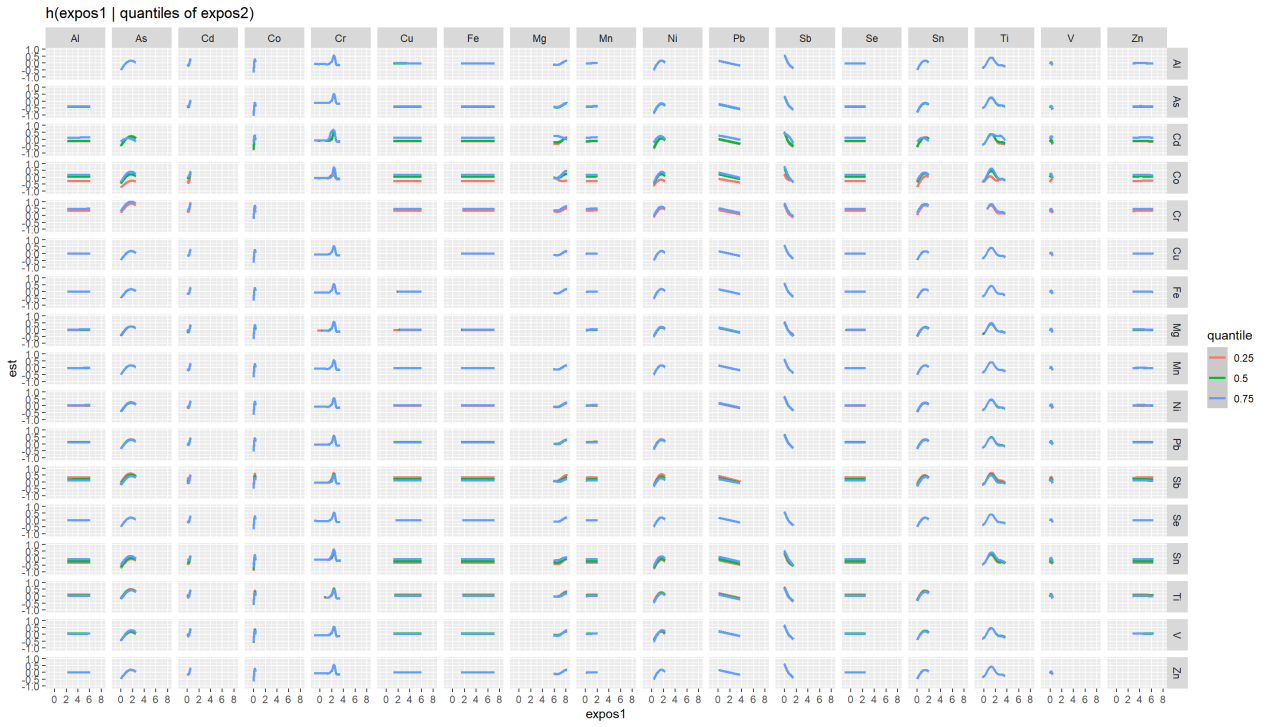


Fig. S2 Bivariate exposure-response function associated with mixed metal exposure and thyroid cancer.
