# Supplementary material for: Association between multi-metal co-exposure and thyroid cancer risk in Shanxi, China: A case-control study
Source: PLoS One. 2026 Jan 23;21(1):e0334872. doi: 10.1371/journal.pone.0334872 (PMC12829857; doi:10.1371/journal.pone.0334872)
Supplement: S1 Table — (DOCX) [file pone.0334872.s003.docx]

Supplementary Table S1. Analytical performance of measured metals: limits of detection (LOD), quantitation (LOQ), detection rates, and precision

| Metals | LOD (µg/L) | LOQ (µg/L) | Detection Rate (%) | Intra-assay CV (%) | Inter-assay CV (%) |
| --- | --- | --- | --- | --- | --- |
| Mg | 300.00 | 1000.00 | 100 | 3.2 | 5.8 |
| Al | 10.00 | 30.00 | 100 | 4.8 | 8.2 |
| Ti | 5.00 | 20.00 | 100 | 6.1 | 5.4 |
| V | 0.05 | 0.20 | 98.7 | 5.5 | 8.8 |
| Cr | 5.00 | 20.00 | 100 | 5.9 | 9.1 |
| Fe | 50.00 | 200.00 | 100 | 4.2 | 7.3 |
| Co | 0.02 | 0.05 | 96.4 | 7.2 | 9.8 |
| Mn | 0.30 | 1.00 | 99.2 | 6.7 | 7.9 |
| Ni | 1.00 | 3.00 | 100 | 6.4 | 8.0 |
| Cu | 20.00 | 50.00 | 100 | 3.9 | 6.7 |
| Zn | 50.00 | 200.00 | 100 | 4.5 | 6.0 |
| As | 0.05 | 0.20 | 100 | 5.3 | 7.6 |
| Se | 3.00 | 10.00 | 100 | 6.9 | 7.3 |
| Cd | 0.10 | 0.30 | 100 | 4.5 | 5.2 |
| Sn | 0.05 | 0.20 | 99.3 | 4.2 | 8.1 |
| Sb | 0.10 | 0.30 | 100 | 6.8 | 6.3 |
| Pb | 0.10 | 0.30 | 100 | 7.1 | 8.6 |
| Tl | 0.03 | 0.10 | 15.9* | 12.5 | 18.8 |

Coefficient of variance, CV
